# Mental and Physical Well-Being and Burden in Palliative Care Nursing: A Cross-Setting Mixed-Methods Study

**DOI:** 10.3390/ijerph19106240

**Published:** 2022-05-20

**Authors:** Susann May, Franziska Gabb, Yuriy Ignatyev, Jana Ehrlich-Repp, Kerstin Stahlhut, Martin Heinze, Matthew Allsop, Henrikje Stanze, Felix Muehlensiepen

**Affiliations:** 1 Center for Health Services Research, Brandenburg Medical School Theodor Fontane, 15562 Rüdersdorf, Germany; franziska.gabb@mhb-fontane.de (F.G.); yuriy.ignatyev@mhb-fontane.de (Y.I.); martin.heinze@immanuelalbertinen.de (M.H.); felix.muehlensiepen@mhb-fontane.de (F.M.); 2 Landesarbeitsgemeinschaft Onkologische Versorgung im Land Brandenburg e.V., 14469 Potsdam, Germany; ehrlich-repp@lago-brandenburg.de; 3 Department of Oncology and Palliative Medicine, Immanuel Klinik Rüdersdorf, 15562 Rüdersdorf, Germany; kerstin.stahlhut@immanuelalbertinen.de; 4 Department of Psychiatry and Psychotherapy, Immanuel Klinik Rüdersdorf, 15562 Rüdersdorf, Germany; 5 Academic Unit of Palliative Care, Leeds Institute of Health Sciences, University of Leeds, Leeds LS2 9JT, UK; m.j.allsop@leeds.ac.uk; 6 Hochschule Bremen, Centre for Nursing Research and Counselling, 28199 Bremen, Germany; henrikje.stanze@hs-bremen.de; 7 Faculty for Health Sciences, Brandenburg Medical School, 16816 Neuruppin, Germany

**Keywords:** palliative care, nurses, stress, wellbeing, work conditions

## Abstract

The working routines in palliative care nursing are associated with a variety of causes of stress with regional and setting-specific differences in Germany. This mixed-methods study aimed to investigate the mental and physical well-being among nurses in German palliative and hospice care and to gain a deeper understanding of procedural and structural aspects that may influence the mental and physical burden in palliative nursing care. The mixed-methods approach combined qualitative interviews, (*n* = 16) an online survey (*n* = 101), and subsequent data validation in a focus group (*n* = 6). Interview and focus group data were analysed using structured qualitative content analysis. Survey data were analysed using descriptive statistics and an explorative quantitative analysis. Moderate to very high levels of stress were reported across all settings, but were highest for nurses in specialized outpatient palliative care settings. Underlying causes of stress related to working conditions in the nursing profession across all palliative care settings were poor working hours, perceived inadequate remuneration, and high demands for documentation. To ensure sustainable high-quality palliative care, adaptations to working conditions that target causes of stress and burden in palliative care nurses are required.

## 1. Introduction

The number of people living with chronic degenerative and disabling diseases has increased, contributing to a rising need for palliative care [1]. Many people at the end of life may benefit from early integration of the patient-centred approach of palliative care, focusing on quality of life and relief of symptoms [2,3,4]. In addition to palliative medical care, high-quality palliative nursing care, which goes far beyond the usual treatment care, is essential for people with advanced disease and their caregivers and family [5].

The working routines in palliative care nursing are associated with a variety of physical, psychological, and social causes of stress. Continuing increases in demands on palliative care nurses are leading to an intensification of work which manifests itself in adverse working conditions, such as very long duties, rotating shifts, and low numbers of staff for each shift [6]. Further organisational challenges additionally contribute to stress, such as carrying out too many non-patient related tasks, time pressure or having to care for too many patients at the same time [7,8]. Emotional challenges were also identified in other studies like facing death [9] or inappropriate expectations of relatives regarding end-of-life care and conflicts with relatives [10]. Overall, nurses are considered to be at a high risk of developing mental illness, with an increased tendency to develop burnout symptoms and an intention to leave their job [6,11] due to symptoms such as sleeping and concentrating disorders, decreased performance capacity, (emotional) exhaustion, and depression [12].

Burnout is a concept that is characterised by depleted personal and/or social resources [13] and has significant consequences for healthcare organisations, the individual, and the patient population [14]. A previous systematic review on the prevalence of burnout among palliative care professionals found that 17% of these healthcare professionals across the countries suffer from burnout [15].

Burnout also influences nurses’ reported quality of care, increases the likelihood of mistakes in care delivery, and decreases patient satisfaction [16,17]. In summary, symptom burden in this study is defined as the subjective, quantifiable prevalence, frequency, and severity of symptoms placing a physiologic burden and producing multiple negative, physical, and emotional responses [18]. COVID-19 has led to further intensification for palliative care workers globally [19,20]. For example, in community settings during the pandemic, general practitioners in the UK were able to move to predominantly remote consultations but community nursing teams had to continue face-to-face care for patients [21,22].

In Germany, palliative care is sectioned into general palliative care and specialised palliative care. In general palliative care, mostly general practitioners and nursing teams care for terminally ill and dying patients. Specialised palliative care is provided by specialist outpatient palliative care (SOPC) providers, in inpatient hospices (IHC), or palliative care units in hospitals (PCU) [23,24]. In PCUs, the focus is on alleviating symptoms and improving quality of life to allow the critically ill patients to return to their homes and spend their remaining time in their familiar surroundings. IHC are independent residential facilities that focus on people with an advanced life-limiting illness and their relatives with their respective needs. Holistic care and support are provided by full-time and voluntary hospice staff in cooperation with general partitioners. In contrast, SOPC accompanies patients in their familiar surroundings and is available 24 h a day, seven days a week [25].

In the context of German palliative care, there is evidence that the experience of stress differs regionally and can be setting-specific [26,27,28]. There is also international evidence that the experience of stress differs between settings [15]. However, there is only limited literature on the experience of German palliative care nurses. This mixed-methods study aimed to fill the gap with cross-setting and trans-regional data on the mental and physical well-being of nurses in German palliative and hospice care in order to gain a deeper understanding of procedural and structural aspects that may influence burden in palliative nursing care. This study is based on a conceptual framework that elaborates the structure and dynamics of the system in which clinicians work and reveals potential levers for change [29]. This framework represents an approach that demonstrates that processes associated with burnout and professional well-being are complex and occur over time within the context of a multi-level system that influences those processes. There are three interacting system levels—frontline care delivery, health care organization, and external environment—whose characteristics influence the factors contributing to burnout and professional well-being. This framework was adapted to the care provided by palliative care nurses and provides an orientation for categorising the identified causes of stress in order to derive practical implications in a meaningful context, addressing the following research questions:

- What causes of stress do palliative care nurses experience physically and psychologically and how do these affect everyday care?
- How does the experience of stress differ for palliative care nurses across different settings?
- What practical implications can be derived to improve aspects of clinical practice and the work environment to benefit palliative care nurses?

## 2. Materials and Methods

### 2.1. Study Design

**Figure 1:**
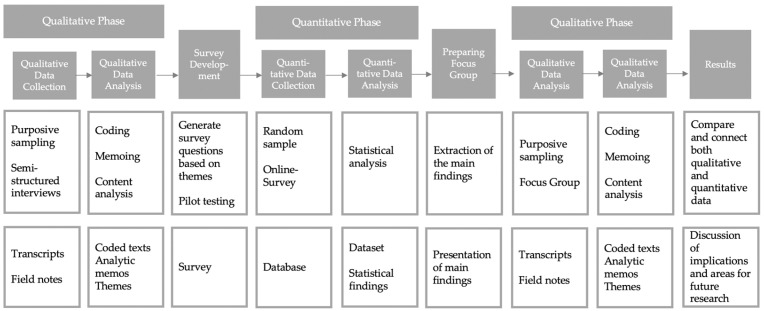
Mixed-methods study design.

To explore the determinants of mental and physical well-being in palliative nursing care, the present study was based on an exploratory mixed-methods approach [30] (Figure 1). It combined qualitative interviews, an online survey, and a focus group. The subsequent survey was used to further explore factors of importance identified in the previous qualitative phase. The ensuing focus group was used to validate the results. Data were collected, analysed, and synthesised in an iterative process. The findings and recommendations of the present study will be aligned with a framework [29].

The study was conducted in compliance with current data protection regulations and the Helsinki Declaration in its current form [31]. All study participants were informed about the research project. Participants of the qualitative research provided written consent. This study was approved by the Ethics Committee of the Brandenburg Medical School Theodor Fontane, Reference ID: E-01-20200511. This manuscript and its reporting align with checklists for the Consolidated Criteria for Reporting Qualitative Research (COREQ) [32] (Supplementary Material S1), the reporting of observational studies (STROBE) [33] (Supplementary Material S2), and reporting of a mixed-methods study (GRAMMS) [34] (Supplementary Material S3).

### 2.2. Expert Interviews

To explore well-being and causes of stress in the different settings of palliative care, semi-structured interviews [35] were conducted. Participants were selected using purposive sampling, specifically, criterion sampling with the aim to include palliative care nurses from different palliative care settings [36]. Thus, the inclusion criteria were the following: working in a palliative care setting (Inpatient Hospice Care (IHC), Palliative Care Unit (PCU) or Specialized Outpatient Palliative Care (SOPC)) and being employed as a nurse for two years to ensure that nurses were firmly established with experience in the context of palliative care nursing. Participants were recruited from healthcare institutions that are clinical partners of the Center for Health Services Research of the Brandenburg Medical School. Eligibility was verified prior to the interview as part of the scheduling of the interview by telephone. The preliminary interview guide was drafted by a professional advisory board for the project, comprising professionals from each palliative care setting included in the study. The interview guide was piloted in the first two interviews. Finally, it was found applicable, as only minor editorial adjustments were necessary. The main topic areas explored were experience of burden in daily routines due to patients, relatives, and work-related factors; and focusing interventions to reduce burdens. To reduce infection risk, the qualitative interviews were conducted via phone from March 2019 to December 2020 by F.M. and S.M. In addition, socio-demographic data were collected, including gender, age, and job position of the interviewees.

### 2.3. Survey

Three researchers (S.M., F.M., F.G.) designed the questionnaire based on the results of the expert interviews. Data from the qualitative study phase were used to develop a survey instrument for the subsequent quantitative phase of the study. In the first step, themes derived from expert interviews were transformed into items for the survey (S.M). After independent review and modification (F.M., F.G.), comments were discussed in the study group (H.S., J.E.R.) and adopted (S.M., F.M., F.G.). In the third step, three palliative care nurses and two researchers, who were not involved in the study, assessed the survey for readability and ease of use. Overall, they reported that the survey was easy to understand. Minor revisions to refine wording were made accordingly. The final questionnaire comprised four questions: two questions on the causes of stress and activities that may reduce stress (the response options emerged from the themes drawn from the qualitative data), and two questions on the overall rating of the psychological and physical stress experience, with the following response options: very high, high, moderate, low and very low. Finally, a section regarding socio-demographics was added. The inclusion criteria for the online survey were (1) being a qualified nurse; (2) ≥18 years; and (3) working in a palliative care setting in Germany. Sampling was based on a non-probability, voluntary approach by involving two professional associations (the German Association for Palliative Medicine and the Association for Oncological Care Brandenburg e.V.), which distributed the link to the online survey. The data collection was conducted from 19 January to 28 February 2022. Questionnaire responses exported from the survey were imported into SPSS. The analysis included descriptive statistics: quantities, percentages, and median scores.

An additional exploratory quantitative analysis was conducted in four steps. Firstly, categorical scales were converted to ordinal scales. For the questions in the section “Stress-reduction interventions in daily work”, the responses “No” and “Yes” were coded as “0” and “1”, respectively. According to the hypotheses—deducted from the qualitative findings—that stress increases from the specialised palliative home care to the palliative care unit and then to the inpatient hospice, the three settings of palliative care were coded as “1”, “2” and “3”, respectively, and used as an ordinal variable called “palliative care service”. In a second step, linear trends of mental and physical stress, as well as current causes of stress, were explored using the Jonckheere–Terpstra test with a Monte Carlo simulation (1000 samples) [37] of the R-package DescTools [38], one-tailed (alternative hypothesis: increasing). The hypothesis was that these groups were arranged in a certain order, in the expectation that the participants from the services would have a higher level of stress and would be more burdened by stress causes in the following direction: inpatient hospice < palliative care unit < palliative home care. In a third step, a linear trend of stress reduction interventions was examined across nurses from the above palliative services, using the Cochran–Armitage test for trend [39] of the R-package DescTools [38], also one-tailed, but in two directions (alternative hypotheses: increasing and decreasing). Testing the two alternative hypotheses was based on the expectation that different stress reduction interventions have different meanings in the group with relatively high and low levels of stress. All analyses based on R packages were run in R version 1.4.1103 (R Core Team, 2021).

### 2.4. Focus Group

The aim of the focus group was the communicative validation of the survey results and the identification of interpretative approaches. The focus group was conducted on 23 March 2022. The recruitment of participants in the focus group followed an ad hoc voluntary approach. All members of the psycho-oncology and palliative care working group of the Brandenburg Medical School were contacted via email and invited to participate in the focus group. The inclusion criterion for the focus group was being a stakeholder in palliative care delivery in Germany. The focus group began with the presentation of the survey results followed by a discussion with participants.

### 2.5. Qualitative Content Analysis

Both the expert interviews and the focus group were audio-recorded and transcribed verbatim. Qualitative analyses of the expert interviews and the focus group were performed iteratively by two health researchers (S.M., F.G.), based on Kuckartz’s structured qualitative content analysis [40] using MAXQDA software (Verbi GmbH). Inductive content analysis was used. First, quotes directed by the aim were extracted and condensed into codes. Main categories and subcategories were formed from the codes. Consensus discussions were held continuously in the research group until a common understanding of all the emerging categories was achieved. The application of the category system was validated again by an internal review to ensure traceability, whereby two researchers independently applied the developed category system to the entire data (S.M., F.M.). Data collection and analysis were circular and continued until no substantially new findings emerged and theoretical saturation was reached. For the presentation of the results, representative quotes from the transcripts were selected, translated into English, and included in the manuscript.

## 3. Results

From May 2020 to March 2022, a participatory mixed-methods study on burden in palliative care was conducted, consisting of expert interviews with nurses (*n* = 16), a national online survey (*n* = 101), and one focus group with stakeholders of palliative care delivery in Germany (*n* = 6).

### 3.1. Expert Interviews

In total, 16 interviews were conducted with nurses in palliative care. We included five nurses of IHC, five nurses of PCU, and six nurses of SOPC. One nurse had not undergone palliative care training. The group of interviewees consisted of five head nurses and eleven nurses. The mean age of the participants was 48.1 years (Table 1). The interviews lasted between 42 and 77 min in duration. The mean duration of interviews was 55 min.

We identified three main categories of causes of stress in the different settings of palliative care: (i) patient-related, (ii) caregiver-related, and (iii) working conditions specific to the setting.

#### 3.1.1. Patient-Related Causes of Stress

The nurses working across all palliative care settings reported that stress is caused by the intensity of the relationships with patients and the omnipresence of death and dying. At the same time, however, dying is experienced as a natural process that, while stressful, is part of their jobs.

*“It’s exhausting, you’re already exhausted and we’re also sad and cry with the people. It’s not that I’m desperate that they die. It’s more natural. When many people die, we are also emotionally involved and exhausted. But we are not desperate. On the contrary, I’m really fine. So I often say: “I’m happy”.”*

(5_PCU, Pos. 90)

Additionally, the interviewees described that the symptom severity of the patients and the complexity of the conditions have to be met by extensive care, which is perceived as burdensome.

#### 3.1.2. Caregiver Related Causes of Stress

Family members and other related people are part of the patients’ care environment. Comprehensive explanations and detailed instructions for the caregivers are indispensable. However, these can be a source of stress for nurses, especially when opinions or advice by nurses are overridden. Especially in the SOPC, the high expectations of relatives towards nurses’ treatment options were also perceived as burdensome. For example, the relatives anticipate a lot of interventions and expect nursing staff to be able to act immediately and quickly.

*““And why can’t you do anything? You have a whole case full. Look at how she’s breathing.”. Well, the fact that we are always in such a pressure situation to really meet the requirements”*

(11_ SOPC, Pos. 12)

#### 3.1.3. Causes of Stress Related to Working Conditions

We identified the following sub-categories as causes of stress related to working conditions: staff shortages, documentation effort, type of working model, lack of time in daily routines, specificity of nursing activities, and remuneration. However, there are variations between settings of care, which are presented in Table 2.

#### 3.1.4. Interventions Reducing Burdens

In addition to the causes of stress in daily routines, nurses were asked about approaches that may support reductions in stress. Various interventions were identified, with alignment to different settings of palliative care delivery (Table 3).

### 3.2. Survey

The qualitative results were used to develop themes and questionnaire items for the survey. The sample included 101 participants, 87 (86%) of whom were female and 14 (14%) were male. The mean age was 47.9 years (SD 10.4) with a range of 19–67 years. The participants consisted of SOPC nurses (*n* = 44), nurses of PCU (*n* = 30), and IHC nurses (*n* = 27). Demographic characteristics and details of each palliative care setting are summarised in Table 4.

#### 3.2.1. Perceived Mental and Physical Burden

**Figure 2:**
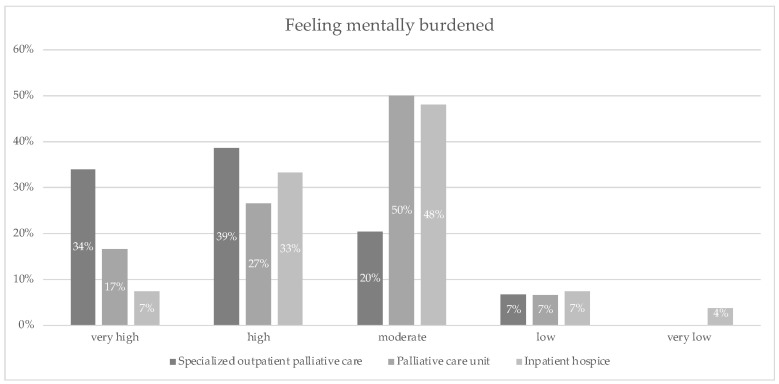
Feeling mentally burdened was measured when asked: “How do you rate your psychological burden at the moment?”; the response options that participants could choose from were: very high, high, moderate, low, and very low.

Perceived stress was measured with the subjective rating of mental burden. Nurses in SOPC reported very high (34%) or high (39%) mental burden. In contrast, about half of the nurses in PCU (50%) and IHC (48%) reported a moderate level of mental burden. The results are displayed in detail in Figure 2.

**Figure 3:**
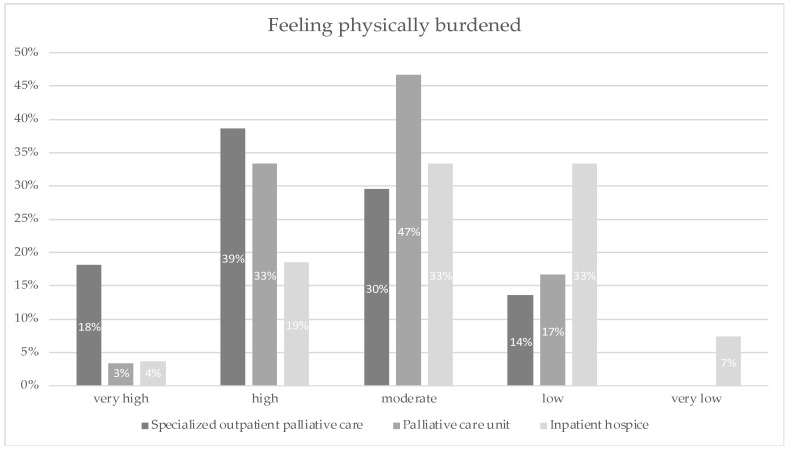
Feeling physically burdened was measured when asked: “How do you rate your physical burden at the moment?”; the response options that participants could choose from were: very high, high, moderate, low and very low.

Overall, 57% of the nurses in SOPC care reported very high or high physical burdens. In contrast, 23% of nurses in IHC and 36% of the nurses in PCU stated this. Overall, IHC nurses feel the least physical strain (40%). Detailed results are displayed in Figure 3.

#### 3.2.2. Current Causes of Stress

The reported stress relating to working with patients does not tend to burden the nurses in their daily work. Additionally, the stress caused by relatives indicates a moderate stress experience. There were no differences in the experience of stress in the different settings. However, a rather heterogeneous pattern emerged relating to stress caused by working conditions. The SOPC is highly burdened by six causes: understaffing, documentation effort, recurring overtime, administrative effort, permanent availability, and remuneration. In contrast, high burden was only reported for two items in the setting of PCU: understaffing and documentation effort. In the IHC, no high burden causes were identified (Table 5).

Stress levels assessed for mental (JT = 2128.5, *p* = 0.001) and physical (2191.5, *p* = 0.001) areas differed significantly in the order “inpatient hospice” < “palliative care unit” < “palliative home care”. The differences in this direction were found also for causes of stress (Table 6).

Nurses from SOPC were more likely to desire recognition of palliative care by society than nurses from PCU or IHC (Z = 3.781, *p* = 0.0001). The trend in this direction was observed also for the recognition of palliative care by politicians (Z = −3.272, *p* = 0.0005) and inter-professional collaborations (Z = −1.767, *p* = 0.04), as well as toward the reduction of documentation effort, although it did not reach statistical significance (Z = −1.385, *p* = 0.08). Interestingly, there were opposite linear trends for decreasing desires for statutory regulations for adjusting working time (Z = 2.222, *p* = 0.01), reducing working time (Z = 2.249, *p* = 0.01, and promoting exchange between professional groups (Z = 1.921, *p* = 0.03) in the direction from IHC to PCU and then to SOPC.

#### 3.2.3. Measures Reducing Burden in Different Palliative Care Settings

**Figure 4:**
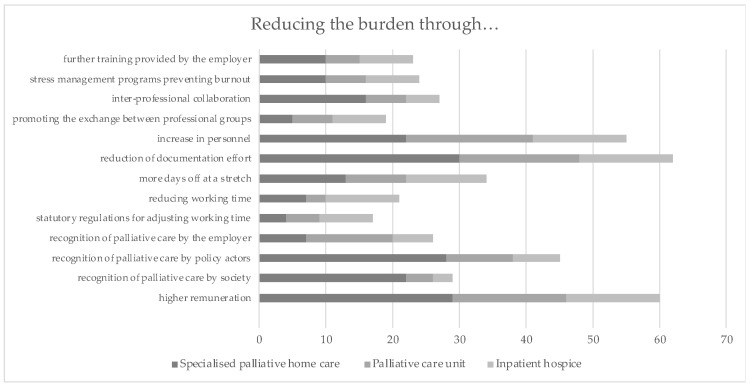
Measures to reduce burdens in different palliative care settings (*n* = 101).

When asked about measures to reduce burden in palliative care, the following four activities were mentioned particularly frequently across the settings: increase in personnel (54%), reduction of documentation effort (61%), recognition of palliative care by policymakers (45%), and higher remuneration (59%). For detailed information, please see Table 7. Once again, a substantial difference can be seen in the response behaviour of the nurses of SOPC compared to the nurses from the other settings. They stated particularly frequently that an improved recognition by society could have a stress-reducing effect (Figure 4).

### 3.3. Focus Group

One focus group was held with six stakeholders of palliative care delivery in Germany including two palliative care physicians, two health researchers, one health insurance representative, and one coordinator of the regional association for oncological care. The focus group revealed a homogeneous spectrum of opinions, with participants confirming the survey results and offering new explanatory approaches.

Participants initially offered explanations about why working in SOPC is perceived as more burdensome than working in IHC or PCU. From the participants’ point of view, nurses in the SOPC staff are “lone fighters” who have to adapt flexibly to new situations with each patient, usually have no one to support them ad hoc, and have only limited access to medical aids.

*“I could imagine that SOPC might feel more burdensome because they have less control. Something we also said before, is that assistive devices are not at hand. When I’m in a hospice or inpatient setting, I’m at home, in a sense, that’s my professional role, I’m here, the patients come to me. I have all the basic requirements here. That’s not the case at the patients’ home. I first have to bring in aids, I have to get accustomed. Then not all the things I suggest are accepted or tolerated. The family decides. They can’t be implemented as easily as in inpatient care”.*

(Focus Group, Health Researcher and Nurse, Pos. 21)

From the participants’ point of view, the aspect of time and timing in SOPC plays a central role in the experience of stress.

*“Of course, I am much more stressed in SOPC alone, because the patients are all in different places. In the hospice or in the clinic, I’m always in the same place”.*

(Focus Group, Coordinator Regional Association for Oncological Care, Pos. 17)

Particularly to relieve nurses in SOPC, employers should take action to support logistics and planning to enable adequate time and flexibility during patient visits.

*“If you look back at the stress factors we mentioned, then you could start there to relieve the burden and claim that there is enough time for each home visit, calculated distances, parking problems, logistical things that can be resolved quite easily”.*

(Focus Group, Palliative Care Physician, Pos. 36)

In addition, the focus group discussion emphasised once again that there is limited understanding of the role of SOPC by the general public, which needs to be addressed and improved.

*“The other thing that came to my mind spontaneously is that SOPC needs another name. That’s like everybody said, we all know what hospice is, it’s a term, SOPC is relatively new and not established yet”.*

(Focus Group, Coordinator Regional Association for Oncological Care. Pos. 50)

Participants mentioned that stress-reducing measures are not only necessary in the SOPC, but they emphasised that a more appreciative attitude and recognition of employers is essential to reduce stress in the professional nursing context.

*“I think that the job profile of caregivers, regardless of the level of qualification, means that in general, not only in SOPC, you are often very dependent on others. You are more of a service provider for the person you are caring for. This can also become a burden if you are not well supported and are in a good employment context. I must honestly admit that I speak from experience”.*

(Focus Group, Health Researcher and Nurse, Pos. 32)

The demand for higher remuneration for palliative care nurses was not fully supported by participants. They reported that this problem should be considered in a differentiated way, as the first motivation for nursing activities is non-financial in nature.

*“I assume that most of them are in this profession due to great intrinsic motivation and that they put up with a lot for it. That it’s not just the compensation, but because they enjoy doing it. And see it as personally fulfilling or resource enhancing”.*

(Focus Group, Health Researcher and Nurse, Pos. 83)

Thus, adjusted remuneration is not the only solution, but currently, the nursing profession lacks attractiveness, so many do not favour the path to this profession.

*“I also believe that it is not only a question of remuneration, because this is also a social problem. Many young people don’t want to go into the nursing profession. They don’t want to work shifts and nights and drive out when the patient is unwell. That’s something that, from my point of view, is very difficult to overcome”.*

(Focus Group, Palliative Care Physician, Pos. 74)

However, the increase in staff could lead to nurses being relieved again and performing their tasks with meaningfulness.

*“The lack of personnel is a decisive factor. If more staff were available, I think that would lead to greater job satisfaction, because it would solve the time problem. Then you would find more satisfaction and also meaningful fulfilment in your job, and you wouldn’t value the other things so much”.*

(Focus Group, Palliative Care Physician, Pos. 65)

## 4. Discussion

This mixed-methods study on mental and physical well-being in palliative care nursing combines findings from qualitative interviews, a survey, and one communicative validating focus group. The multi-perspective approach results indicate that nurses in palliative care experience high levels of stress in their daily practice. In our survey, nurses reported stress across all settings, but particularly in the context of SOPC. We identified distinct causes of stress that appear in the different settings of palliative care delivery investigated. In SOPC, nurses feel very burdened due to recurring overtime, administrative effort, expectations of permanent availability, and perceived inadequate remuneration for work. In PCU, nurses are highly burdened due to understaffing and documentation efforts. In contrast to the other settings, IHC nurses reported a moderate level of stress. While diverse causes were reported, no fundamental structural or process-related factors were identified.

**Figure 5:**
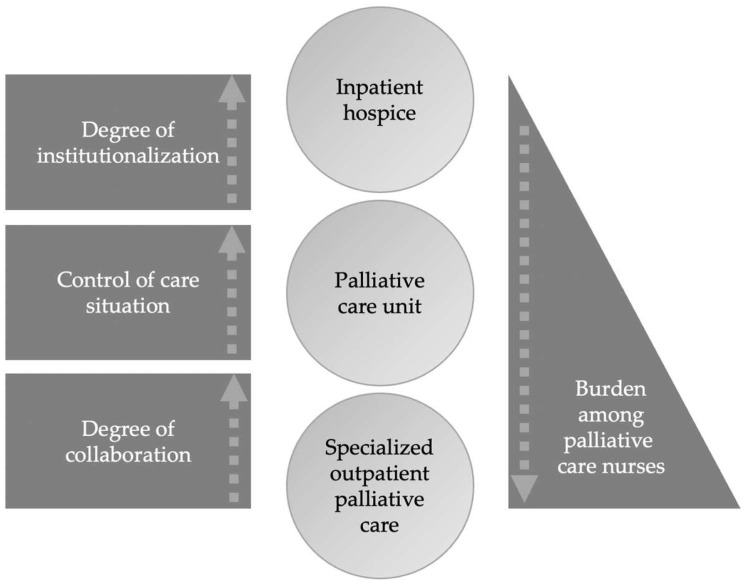
Summarized mixed methods findings.

Combined, the results of all three methodological components of the study suggest a pattern that may underlie stress in the investigated palliative care settings: the higher the degree of collaboration, institutionalization, and ability to control the environment of care delivery, the lesser the perceived stress (Figure 5).

The less control nurses have in their care situation, the more they feel stressed. This applies to SOPC insofar as they can act less flexibly or individually compared to the other settings. Nurses working in this setting are required to continually adapt to new situations and cannot access and utilise medical aids with any immediacy. As in other countries, SOPC in Germany requires the management of complex symptoms and needs to consider the coordination of care, mediate the involvement of relatives, and address issues of death and dying—nurses in this setting are currently navigating this alone, with limited access to resources that are requisite to care delivery [41].

Furthermore, the degree of cooperation seems to influence the perception of stress. In contrast to the IHC and the PCU, SOPC nurses work on their own and cannot use the support of other nurses in their respective care situations. Moreover, the degree of institutionalization influences the experience of stress. The more institutionalized the setting, the less stressed the nurses are. Nurses in more institutionalized settings, such as the IHC or the PCU, with regular work routines, are less stressed.

Our results add to the evidence of high levels of stress in nursing care, which is commonly considered to be caused by the working conditions in the profession [42,43,44]. Specific to palliative care delivery, our findings support the results of other studies, indicating that nurses in SOPC perceive higher strain compared to other settings [45,46]; conceivably, this can be explained by the nature of the activity, which is working in an extremely demanding setting, such as a patient’s home. This phenomenon has also already been described in a previous study [46]: SOPC nurses are considered to be lone fighters who receive no support from other professions in the respective care situation.

High levels of stress among nurses are not only a concern at the individual level, as the poor well-being of providers can negatively influence patient safety [47], its perceived quality (including patient satisfaction), and perceptions of safety [48], which are key dimensions of palliative and hospice care delivery.

The COVID-19 outbreak had an additional impact on palliative care delivery working conditions; over 80% of the participants of a cross-sectional study [49] with palliative care professionals reported being highly or somewhat affected in their ability to continue working in their palliative care role, providing care to non-COVID patients, and issues in staff availability in their workplace. In a previous qualitative sub-study [20] we identified a tension between the palliative care nurses’ perceptions of proper palliative care nursing, in terms of closeness, psychosocial and emotional support, and compliance with infection control measures. Moreover, readjustments in workflows, such as relocation to other wards and changes in infection control, increased the physical and psychological burden among palliative care nurses.

**Figure 6:**
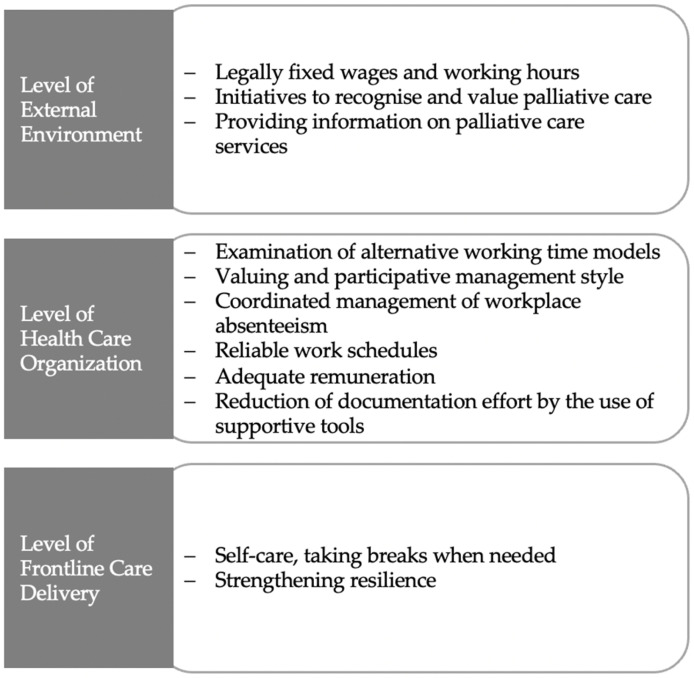
Interventions that could target burden among palliative care nurses.

Based on our findings, we were able to derive interventions that could target improvements in the burden experienced by palliative care nurses (Figure 6).

These suggestions primarily concern the general working conditions in the nursing profession. Adjustment of working time models, adequate remuneration, and the reduction of documentation would not only contribute to improvements in palliative nursing care but also concern the entire nursing profession. The working conditions are interrelated to the pervasive and increasing staff shortages within nursing. If working conditions worsen, fewer trainees enter the nursing profession, causing working hours and documentation effort to increase while the remuneration measured by performance decreases. Good working conditions and especially sufficient time resources are linked to the ability to live out values specific to the profession in everyday work. Concomitant with other studies [50], this is especially important in palliative care as communication and personal attitude are key factors of high-quality care for people living with chronic, progressive diseases.

The results need to be discussed in the light of moral injury, which describes the challenge of simultaneously knowing what care patients need but being unable to provide it due to constraints that are beyond our control [51]. A high correlation of moral injury with burnout has already been shown, suggesting moral injury and burnout are overlapping constructs [52]. Thus, burnout is presumably the symptom of the underlying cause of moral injury [53]. Moral injury allows expressing what burnout is not able to address: being in a double bind and unable to act according to an individual’s ethical beliefs due to structural or hierarchical constraints. This indicates once again that it is not only necessarily about nurses developing individual resilience and providing self-care, but that systemic challenges need to be addressed to prevent burnout and hinder the intention to leave their job to provide high-quality care and healing in the context of health care.

Nursing care is a critical component of palliative and end-of-life care. In 2015, the German Bundestag passed the Hospice and Palliative Care Act to improve the care of the seriously ill and dying nationwide [54]. A variety of measures have been taken, including direct consequences for professionals. However, mental and physical well-being of professionals, especially for nurses, have not been addressed.

Yet, the health status, experiences, and needs of nurses in this situation are rarely investigated. To the best of our knowledge, we have performed the first mixed-methods study on palliative care nurses’ burden in multiple settings of palliative and inpatient hospice care in Germany. The qualitative interviews allowed for an in-depth understanding of the experiences and causes of stress in the day-to-day working routines of palliative nurses. The survey enabled the transfer of individual perspectives assessed in the interviews to a nationwide sample. The survey results were later discussed and validated during focus groups. We recommend this approach, which has repeatedly involved nurses and other stakeholders in palliative care at various levels.

However, there are certain limitations to our study. Due to the high relevance of the topic and defined settings and providers, no specific recruitment strategy (e.g., maximum variation sampling) was pursued, which may have led to self-selection bias in all study modules. In the qualitative interviews, outpatient and volunteer hospice services, which to our knowledge are currently very limited in Germany, were not represented. The online survey was advertised mainly through two professional societies (DGP/LAGO e.V.), hence, it may have been sent to people particularly engaged and digitally savvy who had the resources to complete an online survey. Lastly, the recruitment of participants in the focus group followed an ad hoc approach. Only palliative providers from one region in Germany and nurses from limited settings were able to contribute. Furthermore, our empirical research was implemented after the outbreak of the COVID-19 pandemic. This may have led to a particularly strong experience of stress. The study did not investigate the role of social support in stress and burnout prevention as these aspects were not mentioned in the qualitative interviews and, thus, were not included in the survey. This might be a limitation of our study, which is rooted in the exploratory approach.

Our study highlights the need to undertake further research on the mental and physical well-being of palliative care providers. Firstly, we consider long-term monitoring of stress in palliative care to be highly relevant, which is why we encourage prospective studies on this topic. In addition, the influence of stress on the quality of palliative care and the safety of patients with chronic, progressive illnesses and their relatives requires further investigation. Social support from colleagues and managers could be another useful focus of future research to determine the extent to which they might influence the mental and physical well-being of palliative care nurses. In addition to pure research projects, the implementation of our suggestions to relieve the burden of palliative care nurses would be highly recommended, for example, in the context of a (municipal) pilot project with approaches of the Magnet Hospital Concept [55], in which day-to-day care is organized in a participatory framework under the evaluation of the quality of care and health economic factors.

## 5. Conclusions

Nurses in German palliative care report high levels of burden. Nurses in SOPC report particularly high levels of stress, alongside reported moderate-to-high levels of stress by nurses in PCUs and IHC. The high level of stress is not primarily rooted in the care of patients with chronic, progressive illnesses and their relatives, but due to working conditions including poor working hours, poor remuneration and high documentation effort. To counteract the shortage of qualified staff and ensure high-quality palliative care, working conditions must be improved quickly. Future research should explore the wider acceptability and feasibility of the proposed approaches to reducing stress that were derived from the participants involved in this study.

## Figures and Tables

**Table 1:** Expert interviews sample characteristics.

#	Age	Gender	Setting	Education	Position
1	49	Female	IHC	Nurse with palliative care training	Nurse
2	49	Female	PCU	Nurse with palliative care training	Head nurse
3	27	Female	PCU	Nurse with palliative care training	Nurse
4	54	Female	PCU	Paediatric nurse with palliative care training	Nurse
5	54	Female	PCU	Paediatric nurse with palliative care training	Nurse
6	27	Female	IHC	Nurse with palliative care training	Nurse
7	46	Female	IHC	Nurse with palliative care training	Nurse
8	55	Female	IHC	Nurse with palliative care training	Nurse
9	37	Female	SOPC	Geriatric nurse with palliative care training	Head nurse
10	41	Female	SOPC	Paediatric nurse with palliative care training	Head nurse
11	60	Female	SOPC	Nurse with palliative care training	Nurse
12	58	Female	SOPC	Nurse with palliative care training	Head nurse
13	60	Female	SOPC	Nurse with palliative care training	Nurse
14	51	Female	SOPC	Geriatric nurse with palliative care training	Head nurse
15	60	Female	PCU	Nurse with palliative care training	Nurse
16	42	Female	IHC	Geriatric nurse	Nurse

**Table 2:** Sub-categories of burden due to working conditions.

	PCU	IHC	SOPC
Staff shortages	Participants reported strain due to the lack of staff, as staff frequently changed between wards of the hospital.	Furthermore, staff shortages were common due to a lack of qualified staff and, often, the recruitment or temporary cover of other insufficiently qualified personnel.	Not mentioned.
	*“We always have a very large rotation among the wards. Then you just take someone off from one ward to the other. And because of that you almost never have full staff. And that is stressful”* (5_PCU, Pos. 34)	*“Half of them are geriatric nurses because of a lack of staff. And you notice that they are actually very close to their limits.”* (7_ IHC, Pos. 16)	Not mentioned.
Documentation effort	Documentation is perceived as burdensome because it is seen as hindering the ability of nurses to focus on the delivery of quality care.	Whilst recognition of the importance of documentation was acknowledged, it was seen as a time-consuming and unwelcome deviation of nurses away from the delivery of patient care.	Not mentioned.
	*“A big burden is documentation. It is the be-all and end-all. So if you don’t document, you haven’t done it. Of course, that takes up most of our time, that’s quite clear.”* (2_PCU, Pos. 32)	*“We try to reduce it [the documentation], but despite it all, of course, documentation has to be done. And in such times, things that we would otherwise like to do with the guests are neglected. Even going for a walk, organising other things or something like that, that can’t happen because you don’t have the energy.”* (8_ IHC, Pos. 42)	Not mentioned.
Organisation of work	Nurses reported stress because they have too few days off in a row.	Nurses reported stress due to shift patterns, being unable to have an adequate amount of time when working in a three-shift system.	In the context of outpatient care, nurses reported that they felt pressured to be constantly available and responsive to patients.
	*“These many shifts. We sometimes have ten shifts, all in a row. One day off, that’s just exhausting. You don’t feel like it anymore, that’s how I feel at the moment. I just don’t want to do anything anymore at the moment, I don’t know myself like that at all.”* (4_ PCU, Pos. 28)	*“I’ve noticed that there’s little time left in the full-time, three-shift system to do things that give me enough balance. Meeting friends, digging in the garden, whatever, reading. So there was too little time. And I know from my previous hospice years that if you have three days off at a stretch or something, you switch off in a completely different way and come back with much more energy.”* (8_ IHC, Pos. 62)	*“In SOPC, it is often like: I have to drop everything and leave now. This is sometimes, yes, exhausting.”* (9_ SOPC, Pos. 12)
Lack of time in daily routines	Due to lack of time, care cannot be provided adequately.	Not mentioned.	Lack of time for intensive care (physical as well as psychosocial)
	*“If I don’t have time, I can’t engage very intensively with the patient, which of course also makes me dissatisfied. Lack of time is already a big factor that weighs on you.”* (2_PCU, Pos. 26)	Not mentioned.	*“You do want to take your time: With some, you’re half an hour, with others you’re an hour and a half, or two. You never know. That’s why it’s always difficult and exhausting and you also have to work more sometimes.”* (10_SOPC, Pos. 4)
Nursing activities	Not mentioned.	Not mentioned.	Caring for patients without the support of nursing assistants is perceived as stressful.
	Not mentioned.	Not mentioned.	*“All the nurses who are on duty in the morning and do long hours of washing, or beds, or really do personal hygiene. They don’t have any support. You are alone. That gets to you.”* (11_ SOPC, Pos. 12)

**Table 3:** Interventions reducing burden; x = category was mentioned in the interviews; - = it was not mentioned in the interviews.

	Anchor Quote	PCU *	IHC *	SOPC *
Higher remuneration	*“I simply wish for more recognition. If you will, also financially, so that recognition comes in the form of better pay, because we bear an incredible amount of responsibility at the end of our lives. It doesn’t matter whether I’m dealing with a judge or a lowly cleaner. They are all treated equally well, whether they are private patients or not. I would also like to be treated well, or to be recognised for what we do, for the competence it takes to do a good job.” *(7_ IHC, Pos. 68)	x	x	x
Recognition of palliative care by society	*“I would like to see a much higher recognition of our really versatile services that we strive to provide on a daily basis. Recognition in the financial area, but also in the social appreciation.”* (11_SOPC, Pos. 28)	x	x	-
Recognition of palliative care by politicians	*“You really have to go into politics. We should really go to the parliament and say: “ Please come here, I’ll show you how we provide care for a palliative patient, what it takes in terms of time. What it means to work in palliative care.”* (14_SOPC, Pos. 72)	x	x	x
Recognition of palliative care by the employer	*“Yes, money is one thing, I would say. But the employer could also reward our work differently, just value it more.”* (4_PCU, Pos. 58)	x	-	-
Statutory regulations for adjusting working time	*“Legal regulations on working time. Of course, of course. Yes, it would be nice if there were something like that. Or that they also said every second weekend off.”* (2_PCU, Pos. 126)	x	-	-
Reducing working time	*“And I have a desire to reduce working hours.”* (7_IHC, Pos. 50)	-	x	-
More days off at a stretch	*“That the legislator also really stipulates that after five days, there must be two days off.”* (2_PCU, Pos. 120)	x	x	-
Reduction of documentation effort	*“You could happily reduce the documentation by half.”* (5_PCU, Pos. 54)	x	-	-
Increase in personnel	*“We need staff (laughs). That is really the be-all and end-all, that you get more staff here. That you can eliminate all these burdensome things. That I can really say I can sit down and talk to the patient and don’t have to say I’ll come back in two minutes.”* (2_PCU, Pos. 66)	x	x	x
Promoting the exchange between professional groups	*“Well, an exchange with other professional groups would be great! That would really help at times.”* (14_SOPC, Pos. 160)	-	-	x
Stress management programs preventing burnout	*“And when I hear what palliative work was back then. Mrs. M., for example, has been there for fifteen or twenty years. When she talks like that, you can’t compare it with today’s situation. And that is actually a bit sad. I’ve already noticed that extremely in the four years that I’ve been there. This change in terms of palliative work. Well, in any case, burnout prophylaxis would be very important for me.”* (3_PCU, Pos. 110)	x	-	-
Further training provided by the employer	*“Advanced courses for palliative care nurses, or learning relaxation techniques. That would be something. There are great offers, but they are all expensive. And my employer boasts about its palliative care unit, which is on the website, brochures, everywhere we are always lifted to the skies, but when it comes to further training, nothing happens.”* (4_PCU, Pos. 148)	x	-	-

* Abbreviations: PCU = Palliative Care Unit, IHC = Inpatient Hospice, SOPC = Specialized Outpatient Palliative Care.

**Table 4:** Demographic characteristics.

	SOPC *, n (%)	PCU *, n (%)	IHC *, n (%)	Total, n (%)
Age (years)				
>20	0 (0)	0 (0)	1 (4)	1 (1)
21–30	0 (0)	1 (3)	3 (11)	4 (4)
31–40	8 (18)	7 (23)	6 (22)	21 (21)
41–50	20 (45)	6 (20)	6 (22)	32 (32)
51–60	16 (36)	11 (37)	9 (33)	36 (26)
61–70	0 (0)	5 (17)	2 (7)	7 (7)
Sex				
Total	44 (100)	30 (100)	27 (100)	101 (100)
Female	40 (91)	25 (83)	22 (81)	87 (86)
Male	4 (9)	5 (17)	5 (19)	14 (14)
Professional experience (years)				
>10	3 (7)	4 (9)	9 (20)	16 (16)
11–20	16 (36)	6 (14)	6 (14)	28 (28)
21–30	16 (36)	5 (11)	4 (9)	25 (25)
31–40	8 (18)	12 (27)	6 (14)	26 (26)
<41	1 (2)	3 (7)	2 (5)	6 (6)
Mean	23.5	26.7	20.6	23.7
Median	22.5	30.5	20	24
Palliative care training				
Yes	36 (82)	21 (70)	12 (44)	69 (68)
No	8 (18)	9 (30)	15 (56)	32 (32)
Location				
City	7 (16)	20 (67)	13 (48)	40 (40)
Town	16 (36)	8 (27)	3 (11)	27 (27)
Provincial town	14 (32)	1 (3)	2 (7)	17 (17)
Rural area	7 (16)	1 (3)	9 (33)	17 (17)

* Abbreviations: PC = Palliative Care Unit, IHC = Inpatient Hospice, SOPC = Specialized Outpatient Palliative Care.

**Table 5:** Current causes of stress.

	Current Causes of Stress **	Total (*n* = 101)	SOPC * (*n* = 44)	PCU * (*n* = 30)	IHC * (*n* = 27)
		Median	Median	Median	Median
Patient-related burdens	Close relationship with the patients	4	4	4	4
	Omnipresence of death and dying	4	4	4	4
	Symptom burden of the patients	3	3	3	3
Burdens related to relatives	Need for consulting and information of the relatives	3	3	4	4
	Pressure of expectations on the part of relatives	3	3	3	3
Burdens due to working conditions	Documentation effort	2	2	2	3
	Changing personnel	4	4	4	4
	Understaffing	2	2	2	3
	Shift work	4	3	4	4
	Few days off at a time	3	3	3.5	4
	Physical Stress due to nursing activities	3	3.5	3	3
	Recurring overtime	3	2	3	4
	Administrative effort	2	2	2.5	3
	Permanent availability	3	2	3.5	4
	Remuneration of the work	3	2	2.5	4

* Abbreviations: PC = Palliative Care Unit, IHC = Inpatient Hospice, SOPC = Specialized Outpatient Palliative Care. ** 1 = very highly burdened 2 = highly burdened 3 = partly burdened 4 = slightly burdened 5 = not burdened at all.

**Table 6:** Results derived from the explorative quantitative analysis.

Current Causes of Stress	JT	*p*-Value
Close relationship with the patients	2004	0.014
Omnipresence of death and dying	1699	0.392
Symptom burden of the patients	1758	0.266
Need for consulting and information of the relatives	1926.5	0.045
Pressure of expectations on the part of relatives	1754	0.267
Documentation effort	2006	0.015
Changing personnel	1622.5	0.570
Understaffing	2022	0.011
Shift work	1918.5	0.049
Few days off at a time	1902	0.061
Physical stress due to nursing activities	1598	0.646
Recurring overtime	2267.5	0.001
Administrative effort	2012	0.016
Permanent availability	2264	0.001
Remuneration of the work	2001.5	0.017

**Table 7:** Reducing stress in daily work.

Reducing the Burden Through…	Total n, (%)	SOPC *n*, (%)	PCU n, (%)	IHC n, (%)
Higher remuneration	60 (59)	29 (66)	17 (57)	14 (52)
Recognition of palliative care by society	29 (29)	22 (50)	4 (13)	3 (11)
Recognition of palliative care by policy actors	45 (45)	28 (64)	10 (33)	7 (26)
Recognition of palliative care by the employer	26 (26)	7 (16)	13 (43)	6 (22)
Statutory regulations for adjusting working time	17 (17)	4 (9)	5 (17)	8 (30)
Reducing working time	21 (21)	7 (16)	3 (10)	11 (41)
More days off at a stretch	34 (34)	13 (30)	9 (30)	12 (44)
Reduction of documentation effort	62 (61)	30 (68)	18 (60)	14 (52)
Increase in personnel	55 (54)	22 (50)	19 (63)	14 (52)
Promoting the exchange between professional groups	19 (19)	5 (11)	6 (20)	8 (30)
Inter-professional collaboration	27 (27)	16 (36)	6 (20)	5 (19)
Stress management programs preventing burnout	24 (24)	10 (23)	6 (20)	8 (30)
Further training provided by the employer	23 (23)	10 (23)	5 (17)	8 (30)

## Data Availability

All data relevant to the study are included in the article. For further questions regarding the reuse of data, please contact the corresponding author (susann.may@mhb-fontane.de).

## Supplementary Materials

The following supporting information can be downloaded at: https://www.mdpi.com/article/10.3390/ijerph19106240/s1, Supplementary Material S1: COREQ; Supplementary Material S2: STROBE, Supplementary Material S3: GRAMMS.

## Abbreviations

PCU	Palliative Care Unit
IH	Inpatient Hospice
SOPC	Specialized Outpatient Palliative Care
