# Supplementary material for: Mental and Physical Well-Being and Burden in Palliative Care Nursing: A Cross-Setting Mixed-Methods Study
Source: Int J Environ Res Public Health. 2022 May 20;19(10):6240. doi: 10.3390/ijerph19106240 (PMC9141775; doi:10.3390/ijerph19106240)
Supplement: Supplementary file 1 [file ijerph-19-06240-s001.zip › Supplementary Material S1_COREQ.pdf]

Consolidated criteria for reporting qualitative studies (COREQ): 32-item checklist

Developed from:
Tong A, Sainsbury P, Craig J. Consolidated criteria for reporting qualitative research (COREQ): a 32-item checklist for interviews and focus groups. *International Journal for Quality in Health Care*. 2007. Volume 19, Number 6: pp. 349 – 357

Topic	Item No.	Guide questions/description	Reported on Page #
**Domain 1: Research team and reflexivity**			
Personal Characteristics			
Interviewer/facilitator	1	Which author/s conducted the interview or focus group?	Author information
Credentials	2	What were the researcher's credentials? E.g. PhD, MD	Author information
Occupation	3	What was their occupation at the time of the study?	Author information
Gender	4	Was the researcher male or female?	Author information
Experience and training	5	What experience or training did the researcher have?	N/A
Relationship with participants			
Relationship established	6	Was a relationship established prior to study commencement?	3
Participant knowledge of the interviewer	7	What did the participants know about the researcher? e.g. personal goals, reasons for doing the research	6
Interviewer characteristics	8	What characteristics were reported about the inter viewer/facilitator? e.g. Bias, assumptions, reasons and interests in the research topic	N/A
**Domain 2: study design**			
Theoretical framework			
Methodological orientation and Theory	9	What methodological orientation was stated to underpin the study? e.g. grounded theory, discourse analysis, ethnography, phenomenology, content analysis	4
Participant selection			
Sampling	10	How were participants selected? e.g. purposive, convenience, consecutive, snowball	3
Method of approach	11	How were participants approached? e.g. face-to-face, telephone, mail, email	3
Sample size	12	How many participants were in the study?	5
Non-participation	13	How many people refused to	N/A

		participate or dropped out? Reasons?	
Setting			
Setting of data collection	14	Where was the data collected? e.g. home, clinic, workplace	3
Presence of non-participants	15	Was anyone else present besides the participants and researchers?	N/A
Description of sample	16	What are the important characteristics of the sample? e.g. demographic data, date	5
Data collection			
Interview guide	17	Were questions, prompts, guides provided by the authors? Was it pilot tested?	3
Repeat interviews	18	Were repeat interviews carried out? If yes, how many?	N/A
Audio/visual recording	19	Did the research use audio or visual recording to collect the data?	4
Field notes	20	Were field notes made during and/or after the interview or focus group?	N/A
Duration	21	What was the duration of the interviews or focus group?	5
Data saturation	22	Was data saturation discussed?	5
Transcripts returned	23	Were transcripts returned to participants for comment and/or correction?	N/A
**Domain 3: analysis and findings**			
Data analysis			
Number of data coders	24	How many data coders coded the data?	5
Description of the coding tree	25	Did authors provide a description of the coding tree?	6-9
Derivation of themes	26	Were themes identified in advance or derived from the data?	4
Software	27	What software, if applicable, was used to manage the data?	4
Participant checking	28	Did participants provide feedback on the findings?	N/A
Reporting			
Quotations presented	29	Were participant quotations presented to illustrate the themes/findings? Was each quotation identified? e.g. participant number	6-9, 15-16
Data and findings consistent	30	Was there consistency between the data presented and the findings?	6-9, 15-16
Clarity of major themes	31	Were major themes clearly presented in the findings?	6-9, 15-16

Clarity of minor themes	32	Is there a description of diverse cases or discussion of minor themes?	6-9, 15-16
