# Supplementary material for: Mental and Physical Well-Being and Burden in Palliative Care Nursing: A Cross-Setting Mixed-Methods Study
Source: Int J Environ Res Public Health. 2022 May 20;19(10):6240. doi: 10.3390/ijerph19106240 (PMC9141775; doi:10.3390/ijerph19106240)
Supplement: Supplementary file 1 [file ijerph-19-06240-s001.zip › Supplementary Material S3_GRAMMS.pdf]

Good Reporting of A Mixed Methods Study (GRAMMS) checklist

Guideline	Section: page
Describe the justification for using a mixed methods approach to the research question	Study design: 2
Describe the design in terms of the purpose, priority and sequence of methods	Methods: 2-5
Describe each method in terms of sampling, data collection and analysis	Methods: 2-5
Describe where integration has occurred, how it has occurred and who has participated in it	Discussion: 17
Describe any limitation of one method associated with the present of the other method	Discussion: 19
Describe any insights gained from mixing or integrating methods	Discussion: 17

O'Cathain A, Murphy E, Nicholl J. The quality of mixed methods studies in health services research. J Health Serv Res Policy. 2008;13: 92-98.
